# Lack of an association between marital status and survival in patients receiving stereotactic body radiotherapy for early-stage non-small-cell lung cancer

**DOI:** 10.1371/journal.pone.0269463

**Published:** 2022-06-03

**Authors:** Noriko Kishi, Yukinori Matsuo, Hideki Hanazawa, Yusuke Iizuka, Takashi Mizowaki

**Affiliations:** Department of Radiation Oncology and Image-Applied Therapy, Graduate School of Medicine, Kyoto University, Kyoto, Japan; Taichung Veterans General Hospital, TAIWAN

## Abstract

Marital status has been proposed as a promising prognostic factor in many malignancies, including non-small-cell lung cancer (NSCLC). However, its prognostic value is still unclear for individual non-surgical treatments for stage I NSCLC. This study investigated the prognostic value of marital status in patients with early-stage NSCLC treated with stereotactic body radiotherapy (SBRT). Patients with early-stage NSCLC treated with SBRT between January 2003 and March 2014 at our institute were enrolled, and marital status at the time of SBRT was investigated. Propensity score matching (PSM) was applied to reduce potential selection bias between the married and unmarried groups. Two hundred and forty patients (median age 77 years; 152 married, 87 unmarried) were analyzed. The unmarried included higher proportions of the elderly, women, never smokers, and those with decreased pulmonary function compared to the married. PSM identified 53 matched pairs of married and unmarried patients, with no significant difference in patient background parameters. The 5-year overall survival (OS) was 52.8% and 46.9% in the married and unmarried groups, respectively (*P* = 0.26). There was no significant difference in NSCLC death or non-NSCLC death between the two groups (*P* = 0.88 and 0.30, respectively). There was no significant difference in OS between married and unmarried male patients (n = 85, 5-year OS, 52.6% vs. 46.0%; *P* = 0.42) and between married and unmarried female patients (n = 21, 54.5% vs. 50.0%; *P* = 0.44). In conclusion, marital status was not associated with OS in patients receiving SBRT for early-stage NSCLC.

## Introduction

Marriage has protective effects with respect to survival [1]. It can lead to a healthier lifestyle, better sociomoral support, reduced psychological stress, and higher motivation to be healthy [2–4]. An unmarried status, including being widowed, divorced, or single, is associated with increased mental health problems, cardiovascular diseases, coronary heart disease, and stroke-related death [5,6]. Regarding malignancies, a strong association between marital status and participation in cancer screening has been reported, and participation is even higher in married individuals with a participating partner [7]. In married individuals, cancer is detected earlier, while unmarried individuals are more likely to be diagnosed at an advanced stage and/or be untreated despite diagnosis [8,9]. Unmarried cancer patients have decreased overall survival (OS) compared to married cancer patients in many malignancies after adjusting for stage and treatment [10,11].

Several reports have suggested that marital status could be a prognostic factor in patients with non-small-cell lung cancer (NSCLC) [12–14]. Wu et al. reported a propensity score-matched analysis of 70,006 NSCLC patients from the Surveillance, Epidemiology, and End Results (SEER) database [12]. They showed that married patients had an advantage over unmarried patients in terms of OS and cancer-specific survival. In their report, a subgroup analysis of stage I NSCLC patients showed that unmarried patients treated with surgery had a greater risk of overall mortality than married patients that also underwent surgery. A similar tendency was observed among patients who did not undergo surgery. However, the “without surgery” group was heterogeneous, including patients treated with definitive radiotherapy, systemic therapy, palliative treatment, and supportive care. Therefore, the prognostic value of marital status is still unclear for individual non-surgical treatments in stage I NSCLC patients.

Stereotactic body radiotherapy (SBRT) is a standard treatment option for patients with early-stage NSCLC who are medically inoperable or refuse surgery [15,16]. SBRT is minimally invasive and can be performed as an outpatient therapy, which might lead to less need for social support during and after treatment. The patient population receiving SBRT is usually elderly and frail compared to those treated with surgery, where the effect of marital status is unknown. This study aimed to investigate the prognostic value of marital status in patients with early-stage NSCLC treated with SBRT.

## Materials and methods

This study was performed in accordance with the principles of the Declaration of Helsinki (1975, as revised in 2013) and was approved by our institutional review board. Written informed consent was waived because of the retrospective nature of the study.

### Patient selection

Patients who were clinically diagnosed with early-stage NSCLC and treated with SBRT between January 2003 and March 2014 at our institute were enrolled in this retrospective study. Chest computed tomography (CT) was mandatory at the clinical diagnosis; brain magnetic resonance imaging (MRI) and ^18^F-fluorodeoxyglucose positron emission tomography (FDG-PET) were optional. Bronchoscopic biopsy or CT-guided biopsy was performed prior to SBRT. When histological confirmation was unavailable, NSCLC was clinically diagnosed after discussion in a multidisciplinary oncology team including pulmonologists, thoracic surgeons, radiation oncologists, and diagnostic radiologists considering their medical history, imaging findings, and tumor marker tests. Patients with unknown marital status, synchronous primary lung cancer, biologically effective dose (BED) < 100 Gy (with an alpha/beta ratio of 10 Gy), or those who could not be restaged according to the Union for International Cancer Control (UICC) 8^th^ edition were excluded from the analysis.

### Treatment protocol

SBRT was delivered using multiple coplanar and non-coplanar beams. The details of the SBRT procedure have been previously reported [17,18]. The prescribed doses for peripherally located lesions were 48 Gy in 4 fractions to the isocenter, which corresponds to 42 Gy at the planning target volume (PTV) periphery, until March 2014; 56 Gy in 4 fractions to the isocenter, which corresponds to 49 Gy at the PTV periphery, for tumors ≥ 3 cm from June 2009 to March 2014, and 50 Gy in 4 fractions from March 2014 and thereafter. For centrally located lesions, the prescribed dose was 60 Gy in 8 fractions to the isocenter which corresponded to 52.5 Gy at the PTV periphery [19].

At each follow-up visit, physical examinations and imaging studies (chest radiography or CT) were performed every 3–6 months up to the 5th year and 6–12 months thereafter. All patients were assessed using brain MRI and/or FDG-PET/CT when recurrence was suspected.

The following data were retrospectively collected: age, sex, European Cooperative Oncology Group performance status (ECOG-PS), smoking history (current, former, and never smoker), Charlson comorbidity index (CCI) [20], forced expiratory volume in one second (FEV1), T category clinically restaged according to UICC 8^th^ edition, lobe, tumor location (peripheral or central), histology, indications for SBRT (medically operable but refused surgery or inoperable) and familial support other than spouse. Marital status at the time of SBRT was classified as married or unmarried. Unmarried status included widowed, separated/divorced, and single. All deaths were classified as NSCLC or non-NSCLC deaths. OS was defined as the period from the first day of SBRT to the day of death from any cause and was censored on the last day on which the survival status was verified. Toxicities were graded by Common Terminology Criteria for Adverse Events ver. 4.0. Post-recurrence treatment was defined as any surgery, systemic therapy, or radiotherapy delivered after any recurrence identified clinically or radiographically during the follow-up period.

### Statistical analysis

Patient characteristics were compared using the t-test for continuous values and the chi-square test for categorical values. The reverse Kaplan-Meier method for potential follow-up was used to calculate the median follow-up period [21], and the Kaplan-Meier method was used to estimate OS. Differences in the survival curves were compared using log rank test. The incidences of NSCLC and non-NSCLC death were calculated using the cumulative incidence method, and the differences were evaluated using the Fine-Gray test.

The propensity scores were estimated with logistic regression accounting for six covariates: sex, ECOG-PS, smoking status, and T category as categorical values, and age and CCI as continuous values. Propensity score matching (PSM) was performed using the 1:1 nearest neighbor matching method with a caliper of 0.20. P value of less than 0.05 is considered significant.

Statistical analysis was performed using the R version 4.0.2 software package (R Foundation for Statistical Computing, Vienna, Austria), and PSM was performed using the MatchIt package (version 4.2.0).

## Results

**Fig 1:**
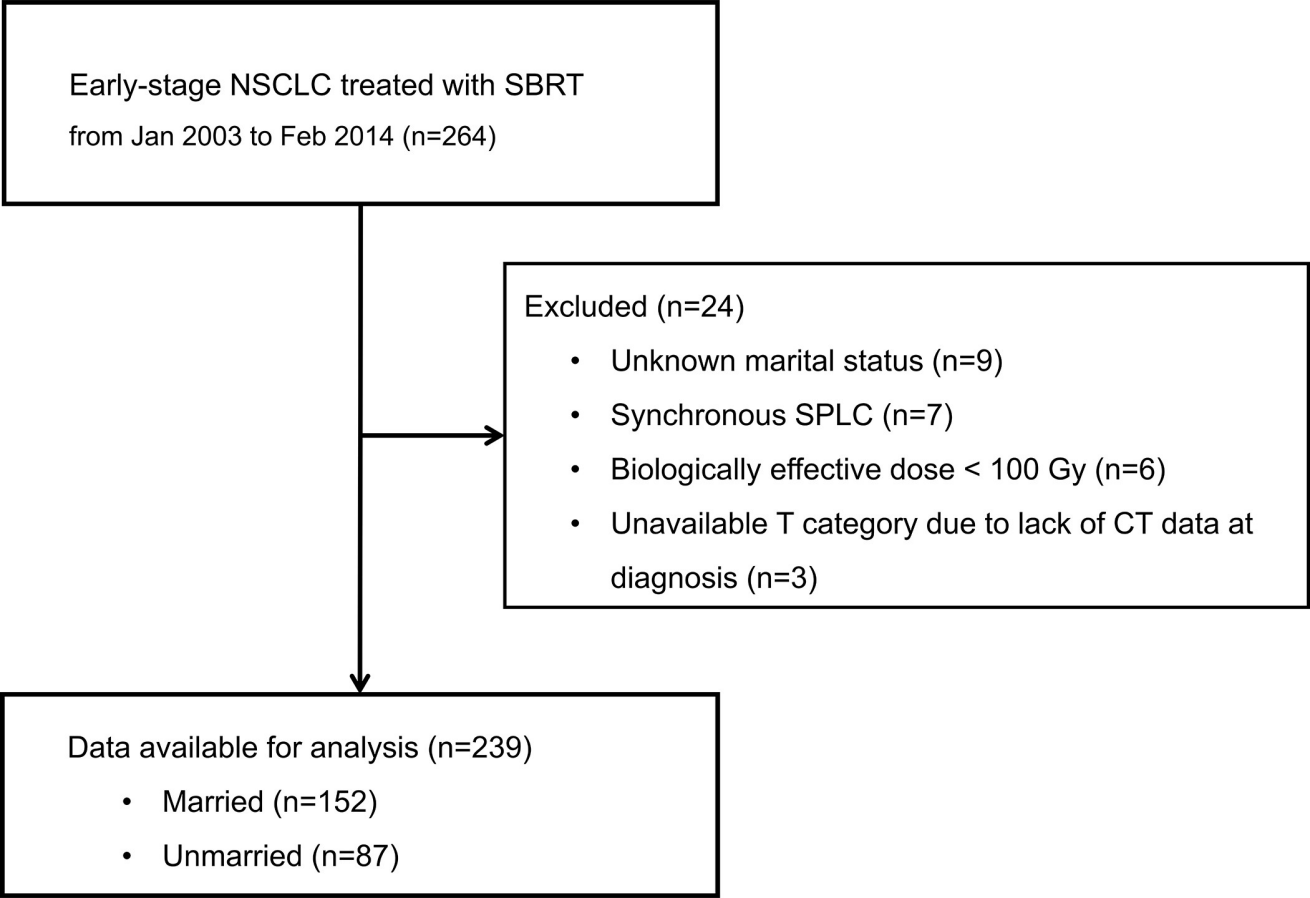
The flow diagram of this study.

Two hundred and sixty-four patients with early-stage NSCLC were treated with SBRT between January 2003 and March 2014 at our institute. Patients with an unknown marital status (9 patients), synchronous double primary cancer (7 patients), biologically effective dose (BED) < 100 Gy (6 patients), and unavailable T category due to lack of CT data at diagnosis (3 patients) were excluded. Two hundred and forty patients were finally included in the analysis (Fig 1).

The median age of the eligible patients was 77 years (range, 56–91). One hundred and eighty-one patients were male and 58 were female. Marital status was “married” in 152 patients and “unmarried” in 87, including 62 widowed, 13 separated/divorced, and 12 single patients. The unmarried group included a higher proportion of elderly patients, women, never smokers, and those with decreased pulmonary function than the married group (Table 1).

**Table 1:** Patient characteristics.

		Overall (n = 239)	Married (n = 152)	Unmarried (n = 87)	*P* value
Age	Mean±SD	77.1±6.9	76.1±7.2	78.7±6.0	0.006
Sex	Female/Male	58/181	14/138	44/43 S/D: 4/9 Widowed: 32/30 Single: 8/4	< 0.001
ECOG-PS	0/1/2+	123/90/26	85/52/15	38/38/11	0.19
Smoking	Current/Former/Never	21/174/44	9/124/19	12/50/25	< 0.001
CCI	0/1–2/≥3	35/120/84	19/75/58	16/45/26	0.29
FEV1*	Mean±SD	1.62±0.61	1.71±0.61	1.46±0.58	0.004
T category	Tis/T1/T2	12/169/58	8/107/37	4/62/21	0.97
Lobe	LUL/LLL/RUL/RML/RLL	61/38/62/21/57	42/23/36/11/40	19/15/26/10/17	0.42
Location	Peripheral/Central	210/29	138/14	72/15	0.10
Histology	Ad/Sq/Others/Unknown	92/70/25/52	52/47/15/38	40/23/10/14	0.21
Indication	Operable/Inoperable	91/148	56/96	35/52	0.70
Familial support other than spouse	Yes/No	224/15	146/6	78/9	0.09

*Abbreviations*: Ad, adenocarcinoma; CCI, Charlson comorbidity index; ECOG-PS, European Cooperative Oncology Group Performance Status; FEV1, forced expiratory volume in one second; LLL, left lower lobe; LUL, left upper lobe; RLL, right lower lobe; RML, right middle lobe; RUL, right upper lobe; S/D, separated/divorced; SD, standard deviation; Sq, squamous cell carcinoma.

* There were no available data on FEV1 in 18 patients.

**Fig 2:**
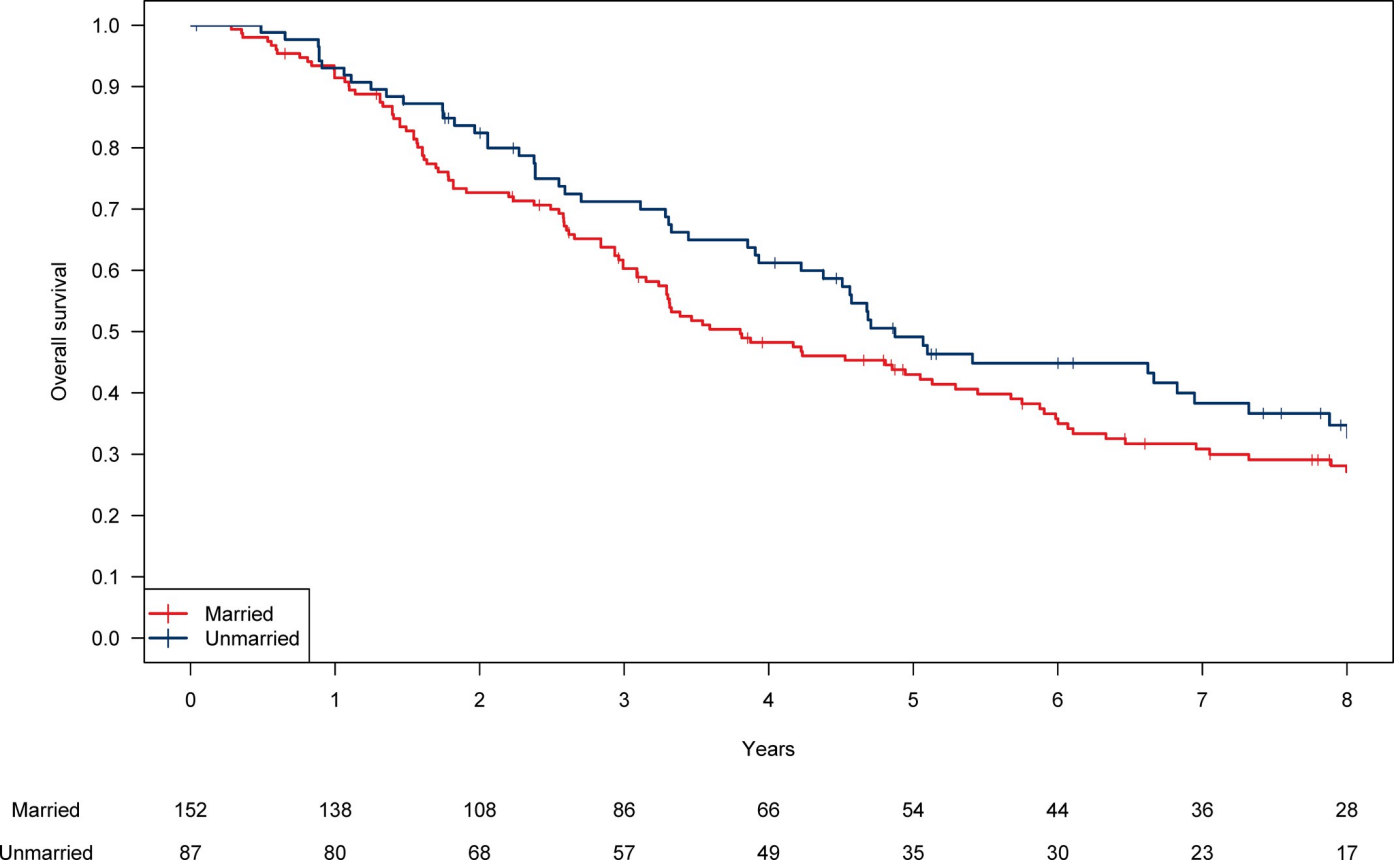
Kaplan-Meier curves for overall survival in the married and unmarried patients for the entire cohort.

With a median follow-up period of 9.0 years (9.0 years for married patients and 9.4 years for unmarried patients), the 5-year OS for all the patients was 45.3% (95% confidence interval [CI], 39.2–52.3%), and the 5-year OS in married patients and unmarried patients was 43.0% and 49.1%, respectively (*P* = 0.12, Fig 2). In widowed, separated/divorced, and single patients, the 5-year OS was 45.6%, 76.2%, and 41.7%, respectively. Among married patients (n = 152), those who had familial support other than spouse (n = 146) showed the 5-year OS of 44.1%, while those who were supported only by spouse (n = 6) showed that of 16.7% (P = 0.13). Among unmarried patients (n = 87), those who had familial support (n = 78) showed the 5-year OS of 51.2%, while those who had no familial support (n = 9) showed that of 33.3% (P = 0.61).

In all the patients, the 5-year cumulative incidence of NSCLC death was 29.9% (31.6% in married patients and 27.0% in unmarried patients; *P* = 0.66). The 5-year cumulative incidence of non-NSCLC death was 24.8% (25.4% in married patients and 23.9% in unmarried patients; *P* = 0.07).

Severe toxicities grade 3 or worse were observed in 15 patients (6.3%): 5 radiation pneumonitis, 4 pneumothorax, 2 pleural effusion, 2 lung infection, and 2 dermatitis.

During the follow-up period, 121 patients developed recurrence (83 married and 38 unmarried patients). Recurrence patterns were as follows: locoregional recurrence only in 57 patients, locoregional recurrence plus distant metastasis in 37 patients, and distant metastasis in 20 patients. The detailed recurrence patterns were unknown in 7 patients. Post-recurrence treatment was delivered to 53 patients, and supportive care was provided to 54 patients without any post-recurrence treatment. The status of post-recurrence treatment was unknown in 15 patients. There was no significant difference in post-recurrence treatment between married and unmarried patients (*P* = 0.23; Table 2).

**Table 2:** The status of post-recurrence treatment in the married and unmarried patients for the entire cohort.

	Any recurrence (N = 121)	Married (n = 83)	Unmarried (n = 38)	*P* value
Best supportive care	53	32	21	0.23
Unknown	15	11	4	
Any post-recurrence treatment	53	40	13	
Surgery/Systemic therapy/Radiotherapy ^†^	11/31/27	10/23/22	1/8/5	

^†^The patients who received multiple post-recurrence treatments during the follow-up period were included.

**Fig 3:**
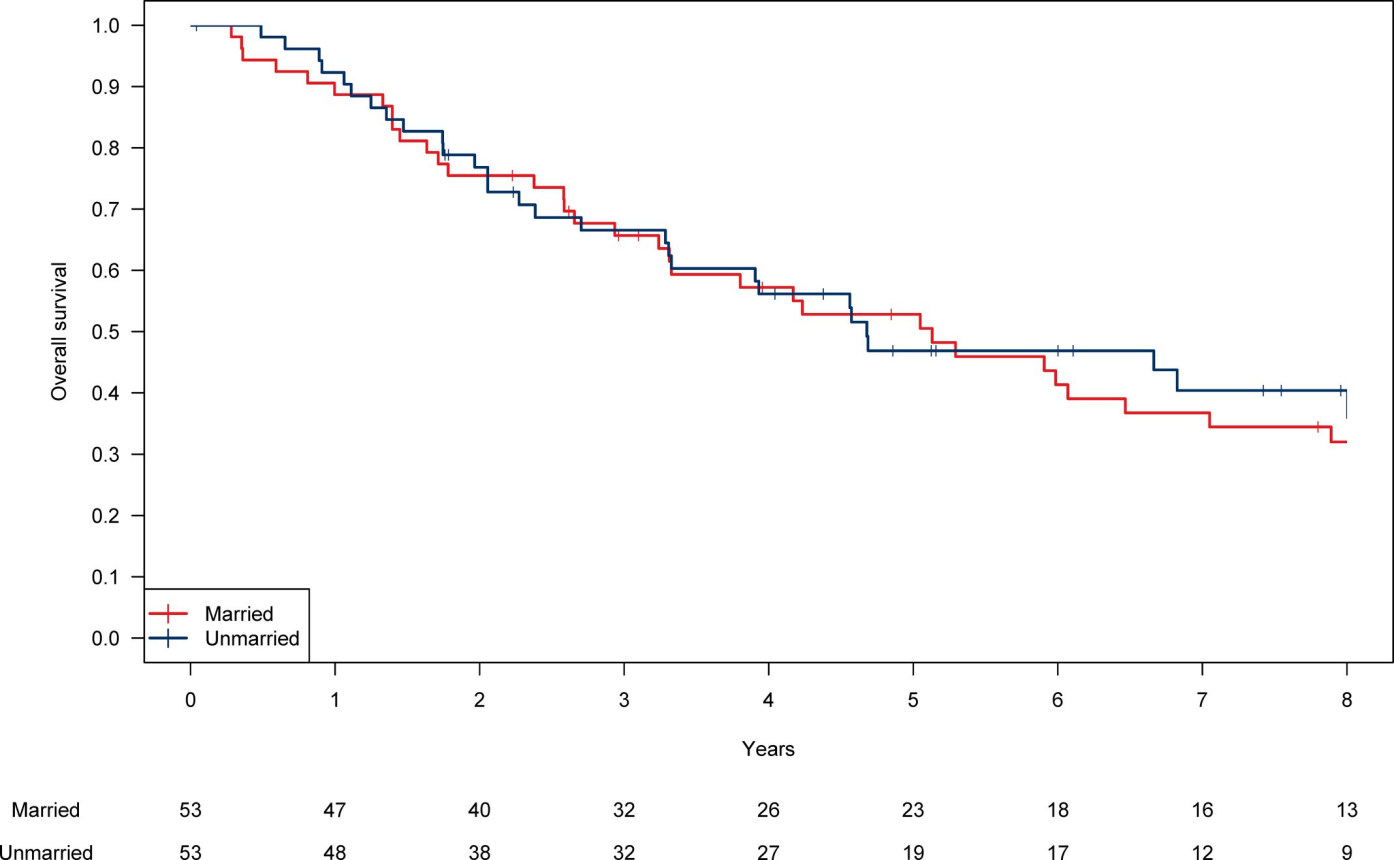
Kaplan-Meier curves for overall survival in the married and unmarried patients for the matched cohort.

PSM identified 53 matched patients from the married and unmarried groups. There was no significant difference in patient background, including six covariates between married and unmarried individuals (Table 3). The 5-year OS in the married and unmarried patients was 52.8% and 46.9%, respectively, with no significant difference (*P* = 0.26, Fig 3).

**Table 3:** Patient characteristics of the matched cohort.

.		Married (n = 53)	Unmarried (n = 53)	*P* value	SMD
Age	Mean±SD	78.1±6.8	78.5±6.4	0.78	0.054
Sex	Female/Male	11/42	10/43	1.00	0.047
ECOG-PS	0/1/2+	27/22/4	27/21/5	0.94	0.071
Smoking	Current/Former/Never	2/41/10	5/39/9	0.50	0.230
CCI	0/1–2/≥3	8/26/19	7/28/18	0.92	0.080
FEV1^‡^	Mean±SD	1.66±0.63	1.61±0.61	0.39	0.083
T category	Tis/T1/T2	3/12/38	2/11/40	0.86	0.11
Lobe	LLL/LUL/RLL/RML/RUL	7/14/15/5/12	10/10/10/6/17	0.53	0.35
Location	Peripheral/Central	47/6	47/6	1.00	< 0.001
Histology	Ad/Sq/Others/Unknown	17/15/5/16	20/15/9/9	0.34	0.36
Indication	Operable/Inoperable	20/33	20/33	1.00	< 0.001
Familial support other than spouse	Yes/No	50/3	48/5	0.71	0.143

*Abbreviation*: SMD, standardized mean difference. Other abbreviations are the same as in Table 1.

^‡^There were no available data on FEV1 in 9 patients.

In the matched cohort, the 5-year cumulative incidence of NSCLC death was 22.9% (19.9% in married patients and 26.0% in unmarried patients; *P* = 0.92). The 5-year cumulative incidence of non-NSCLC death was 27.1% (27.3% in married patients and 27.2% in unmarried patients; *P* = 0.11).

In the matched patients, there was no interaction effect in OS between sex and marital status (*P* = 0.52). The married and unmarried male patients showed similar OS (n = 85, 52.6% vs. 46.0% at 5 years; *P* = 0.42). There was also no significant difference in OS between married and unmarried female patients (n = 21, 54.5% vs. 50.0% at 5 years; *P* = 0.44).

## Discussion

To the best of our knowledge, this is the first study to investigate the prognostic value of marital status in patients with early-stage NSCLC treated with SBRT. Our study showed that marital status was not significantly associated with OS in a cohort consisting of patients receiving SBRT for stage I NSCLC. This study indicates that SBRT might be a treatment option with equal therapeutic potential irrespective of marital status. This is contrary to a previous report on patients treated with surgery for stage I NSCLC [12], where the effect of marital status was evident.

We hypothesized that the following three differences between SBRT and surgery explain the non-significant association between OS and marital status. First, patients treated with SBRT were older than those treated with surgery. The matched cohort in the present study consisted of a high percentage of elderly patients (≥ 80 years, 40%; 70–79 years, 45%; 60–69 years, 13%; < 60 years, 2%) compared with previous reports on surgical cases (≥ 80 years, 17%; 70–79 years, 30%; 60–69 years, 30%; < 60 years, 23%) [12]. The protective effect of marriage, which refers to the association of marriage with a healthier life, is greater for young people than elderly people according to a report based on the Medicare Health Outcome Survey [22]. Even in younger patients, the impact of marital status is attenuated if the prognosis is limited. An ancillary analysis of data from NRG Oncology/RTOG 9704 reported that, in patients with resected pancreatic cancer (median age, 65 years), the association between marital status and OS was not significant (median OS, 1.5 and 1.4 years in married and unmarried patients, respectively) [23]. Second, SBRT requires less social support than surgery. Patients are usually supported, physically, socioeconomically, and mentally, by their spouses or relatives during and after the treatment period. The absence of such support negatively affects survival in unmarried patients treated with surgery. However, SBRT does not cause such a negative effect because it is minimally invasive and requires less hospital care or support than surgery [24,25]. Third, there was a difference in treatment preference between SBRT and surgical patients. As mentioned above, unmarried patients are likely to be untreated at the initial diagnosis [8,9]. In surgical cases, marital status might also affect post-recurrence treatment, which was administered to 69–94% of patients with recurrence after surgery [26,27]. Patients treated with SBRT are generally unfit for or reluctant to undergo intensive treatment; therefore, they prefer minimally invasive treatment at recurrence irrespective of their marital status. Post-recurrence treatment was delivered to less than 50% of the patients in our study cohort, with no significant difference between married and unmarried patients. As we did not directly compare SBRT cases and surgical cases, further investigation is needed to confirm our findings.

There is a sex difference in the effects of marriage. Trevisan et al. reported that marital status influences frailty development differently in women and men [28,29]. Unmarried men were at a higher risk of frailty than married men, while a similar trend was not observed in female patients. In the analysis of the Lung Cancer Database Project in Japan, Saito-Nakaya et al. reported that widowed male patients with NSCLC had a higher mortality rate than married male patients with NSCLC, while there was no significant increased risk of death in widowed female patients compared with married female patients, indicating the “Widow/Widower’s effect” [14]. They also reported that there was no significantly increased risk of death in separated/divorced and single patients compared with married patients. In our study, there was no significant sex difference in OS between married and unmarried patients. The percentages of each unmarried status (separated/divorced, widowed, and single) could have influenced the results, although the differences could not be investigated because of the small sample size.

In the US, Varlotto et al. reported that OS was related to Medicaid insurance and income [30]. There is a universal health insurance system that covers all people living in Japan, ensuring that they have access to healthcare services at an affordable cost. We should consider that the unique situation of the health insurance system in Japan may have reduced the effect of socioeconomic support given to married patients, which was observed in the US series.

This study had several limitations. First, it was a retrospective study conducted at a single institution, and it had a limited sample size. As previous reports on the prognostic value of marital status were based on a large database, it is possible that the sample size of this study had insufficient power to detect the difference. Second, the effect of bias cannot be completely ruled out when comparing married and unmarried individuals. Other confounding factors, including socioeconomic status, mental status, and educational level, were not investigated in this study. There was a selection bias in that patients who received SBRT had more social support than those who received no treatment. Moreover, Murray et al. suggested that there would be a potential selection bias, regarded as “marriage selection”, which refers to those who are able to get married because they are healthy, as well as protective effects of marriage [31].

In conclusion, marital status was not associated with OS in patients receiving SBRT for early-stage NSCLC and may not need to be a focal consideration in patient management for this condition.

## Supporting information

S1 File (CSV)
